# Author Correction: Graphene active sensor arrays for long-term and wireless mapping of wide frequency band epicortical brain activity

**DOI:** 10.1038/s41467-021-23078-z

**Published:** 2021-04-30

**Authors:** R. Garcia-Cortadella, G. Schwesig, C. Jeschke, X. Illa, Anna L. Gray, S. Savage, E. Stamatidou, I. Schiessl, E. Masvidal-Codina, K. Kostarelos, A. Guimerà-Brunet, A. Sirota, J. A. Garrido

**Affiliations:** 1 Catalan Institute of Nanoscience and Nanotechnology (ICN2), CSIC and BIST, Campus UAB, Bellaterra, 08193 Barcelona, Spain; 2 Bernstein Center for Computational Neuroscience Munich, Faculty of Medicine, Ludwig-Maximilians Universität München, Planegg-Martinsried, Germany; 3 Multi Channel Systems (MCS) GmbH, Reutlingen, Germany; 4 Instituto de Microelectrónica de Barcelona, IMB-CNM (CSIC), Esfera UAB, Bellaterra, Spain; 5 Centro de Investigación Biomédica en Red en Bioingeniería, Biomateriales y Nanomedicina (CIBER-BBN), Madrid, Spain; 6 Nanomedicine Lab, National Graphene Institute and Faculty of Biology, Medicine & Health, University of Manchester, Manchester, UK; 7 Division of Neuroscience and Experimental Psychology, School of Biological Sciences, Faculty of Biology, Medicine and Health, University of Manchester, Manchester, M13 9PT UK; 8 ICREA, Pg. Lluís Companys 23, 08010 Barcelona, Spain

**Keywords:** Neuroscience, Biomedical engineering

Correction to: *Nature Communications* 10.1038/s41467-020-20546-w; published online 11 Jan 2021.

The original version of this article included an incorrectly named funding source “Graphene Flagship”, and omitted the names of GrapheneCore 2 and GrapheneCore3 as funding sources, from the Acknowledgements. This has now been corrected in both the PDF and HTML versions of the Article.

The Acknowledgements now reads “This work has been funded by the European Union’s Horizon 2020 research and innovation program under Grant Agreement No. 732032 (BrainCom) and Graphene Flagship Grant Agreements No. 785219 (GrapheneCore2) and 881603 (GrapheneCore3). The ICN2 is supported by the Severo Ochoa Centres of Excellence program, funded by the Spanish Research Agency (AEI, grant no. SEV-2017-0706), and by the CERCA Program/Generalitat de Catalunya. R.G.C. is supported by the International Ph.D Program La Caixa-Severo Ochoa (Programa Internacional de Becas “la Caixa”-Severo Ochoa). This work has made use of the Spanish ICTS Network MICRONANOFABS partially supported by MICINN and the ICTS “NANBIOSIS”, more specifically by the Micro-NanoTechnology Unit of the CIBER in Bioengineering, Biomaterials, and Nanomedicine (CIBER-BBN) at the IMB-CNM. This work is within the project FIS2017-85787-R funded by the “Ministerio de Ciencia, Innovación y Universidades*”* of Spain, the “Agencia Estatal de Investigación (AEI)”, and the “Fondo Europeo de Desarrollo Regional (FEDER/UE)”. A.S. and G.S. were also supported by Bundesministerium für Bildung und Forschung [grant number 01GQ0440]. R.G.C. acknowledges that this work has been done in the framework of the Ph.D in Electrical and Telecommunication Engineering at the Universitat Autònoma de Barcelona. We thank Eduardo Blanco Hernández for assistance with the preprocessing of the motion tracking data.

