# Supplementary material for: Graphene active sensor arrays for long-term and wireless mapping of wide frequency band epicortical brain activity
Source: Nat Commun. 2021 Jan 11;12:211. doi: 10.1038/s41467-020-20546-w (PMC7801381; doi:10.1038/s41467-020-20546-w)
Supplement: Supplementary file 1 — Supplementary Information [file 41467_2020_20546_MOESM1_ESM.pdf]

Supplementary Materials

Graphene active sensor arrays for long-term and wireless mapping of wide frequency band epicortical brain activity

R. Garcia-Cortadella[1*], G. Schwesig[2*], C. Jeschke[3], X. Illa[4,5], Anna L. Gray[6], S. Savage[6], E. Stamatidou[6], I. Schiessl[7], E. Masvidal-Codina[4,5], K. Kostarelos[6,1], A. Guimerà-Brunet[4,5], A. Sirota [2†] and J. A. Garrido[1,8†]

[1] Catalan Institute of Nanoscience and Nanotechnology (ICN2), CSIC and BIST, Campus UAB, Bellaterra, 08193 Barcelona, Spain.

[2] Bernstein Center for Computational Neuroscience Munich, Faculty of Medicine, Ludwig-Maximilians Universität München, Planegg-Martinsried, Germany.

[5] Multi Channel Systems (MCS) GmbH, Reutlingen, Germany.

[4] Instituto de Microelectrónica de Barcelona, IMB-CNM (CSIC), Esfera UAB, Bellaterra, Spain.

[5] Centro de Investigación Biomédica en Red en Bioingeniería, Biomateriales y Nanomedicina (CIBER-BBN), Madrid, Spain.

[6] Nanomedicine Lab, National Graphene Institute and Faculty of Biology, Medicine & Health, University of Manchester, Manchester, UK

[7] Division of Neuroscience and Experimental Psychology, School of Biological Sciences, Faculty of Biology, Medicine and Health, University of Manchester, Manchester M13 9PT, United Kingdom

[8] ICREA, Pg. Lluís Companys 23, 08010 Barcelona, Spain.

* These authors contributed equally to this work

[†] Corresponding author: joseantonio.garrido@icn2.cat (J.A.G.), sirota@biologie.uni-muenchen.de (A.S.)

S1. Specifications of wireless recording system

The wireless system developed by Multichannel systems (MCS) to bias and record from the graphene active sensor arrays consists of two amplification stages followed by a multiplexer to select the output from the first (DC-channel) or second stage (AC-channel). Subsequently the signals are digitized and transmitted in the 2.4GHz band to the receivers (W2100-RE-AO from MCS). The communication between receivers and the computer is done by the interface board (MCS-IFB-in-vitro from MCS). The software used to communicate with the interface board is the commercial software Multi Channel Experimenter. To control the bias conditions V_{gs} and V_{ds} of the active graphene sensors a specific software has been developed, which also allows to quickly extract the $I_{ds} - V_{gs}$ needed to control changes in the charge neutrality point (CNP) of the graphene transistors. The following table summarizes the technical specifications of the wireless system. Fig. S1 shows the dimensions of the wireless headstage, which is connected to a flexible PCB (orange component). The flexible PCB is connected to the headstage on one side and to a PCB containing a zero-insertion-force (ZIF) connector on the other side. The connection between the flexible and rigid PCBs is done by a Molex connector. The flexible PCB eases the alignment of the neural probe on the desired location on the cortex.

DC-Channel count	8
AC-Channel count (with DC enabled)	64 (56)
Input Frequency Range AC (DC)	0.15 Hz – 5 kHz (DC – 7.35kHz)
Maximum input current AC (AC)	+/- 11.5 µA (+/- 115 µA)
Gain AC (DC)	120000 (12000)
Wireless transmission range	5m (depending on environment 10-15m)
OpAmps consumption	52.8mA
ADCs (at Fs = 20kHz) consumption	16mA
RF-Chips consumption	116mA
Battery 3.7V/700mAh duration	~6h
Battery 3.7V/700mAh charging time	~1h
Battery 3.7V/700mAh weight	15g
Headstage weight	7.9g
Flex-PCB weight	0.5g
Weight of 3D printed base ring, headstage support and protective cap	15g

Table S1. Technical specifications of the wireless system

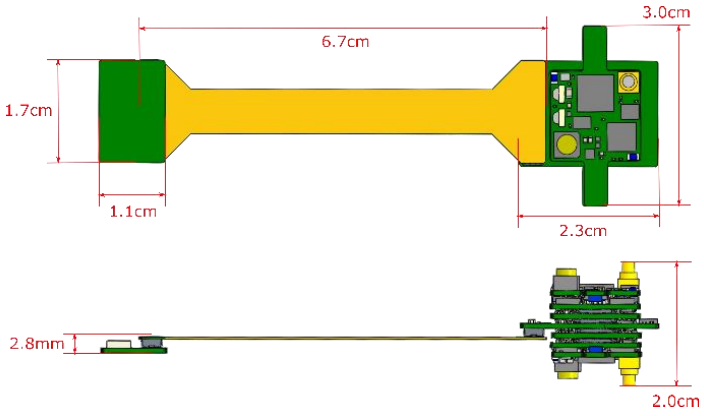

Figure S1. Dimensions of the wireless headstage and connectors. The green component on the left represents a small PCB containing a ZIF connector to the neural probe and a Molex connector to the flexible PCB (orange component). The component on the right represents the wireless headstage

S2. Intrinsic 1/f noise and headstage noise

The equivalent voltage noise spectrum at the gate is shown in Fig. S2A for g-SGFETs connected to DC and AC- coupled channels of the headstage. In the central part of the spectrum (~0.05-10Hz) the 1/f trend corresponding to the intrinsic noise of the g-SGFETs dominates. The intrinsic noise is expected to present a $1/\sqrt{A}$ dependence[1], where A stands for the area of the active part of the graphene channel. Here we have validated that the expected trend holds for the technology presented in this article. Having this validation it is possible to extrapolate the results from this work to devices with different areas. Fig. S2B shows V_{gs-rms} measured for g-SGFETs of different areas fabricated on a flexible PI substrate following the procedure detailed in the Methods section. Note that the values displayed in Fig. 2 of the main text for an area of 100x100 μm^2 match with the trend observed in Fig. S2B.

The intrinsic noise of the g-SGFETs in the infra-slow ($< 0.5Hz$) frequency bandwidth is expected to follow the same 1/f trend as in the rest of the frequency spectrum[2]. However, the low frequency noise from other components of the recording system might contribute significantly in this frequency band. Fig. S2D (top) shows a schematic for the transimpedance amplifier in the first amplification stage, and Fig. S2D (bottom) shows the noise sources in this circuit. The voltage source (V_{ds}) as well as the op-amp present several sources of noise. Fluctuations in V_{ds} are common for all the g-SGFETs and would therefore cause coherent noise in all the channels. Conversely, each channel has an independent op-amp and therefore the noise from this source is expected to be uncorrelated among channels. Table S2 shows the contributions from each of these noise sources, separated between coherent and non-coherent. Fig. S2C shows the coherence between two DC-coupled channels connected to a 2kΩ resistance (red) and to g-SGFETs (black). In the central frequency range, the coherence in the noise of different channels is larger when a resistance is connected instead of the g-SGFETs. The reason is that the intrinsic noise of g-SGFETs is completely uncorrelated among different sensors. At higher and lower frequencies, the coherence increases, presumably due to the effect of quantization noise and correlated noise sources respectively. Finally, Fig. S2E shows the power spectral density measured in DC-coupled channels connected to g-SGFETs in the ISA band (0.005-0.4Hz). The noise below 0.05Hz is shown to clearly deviate from the 1/f trend, indicating a significant contribution of low-frequency noise sources from the headstage. Nevertheless, integrating the noise spectrum in the 0.005-0.05Hz shows that the rms noise increases only by roughly a factor of 2 as compared to the intrinsic noise values shown in Fig. 2 of the main text (see Fig. S2F).

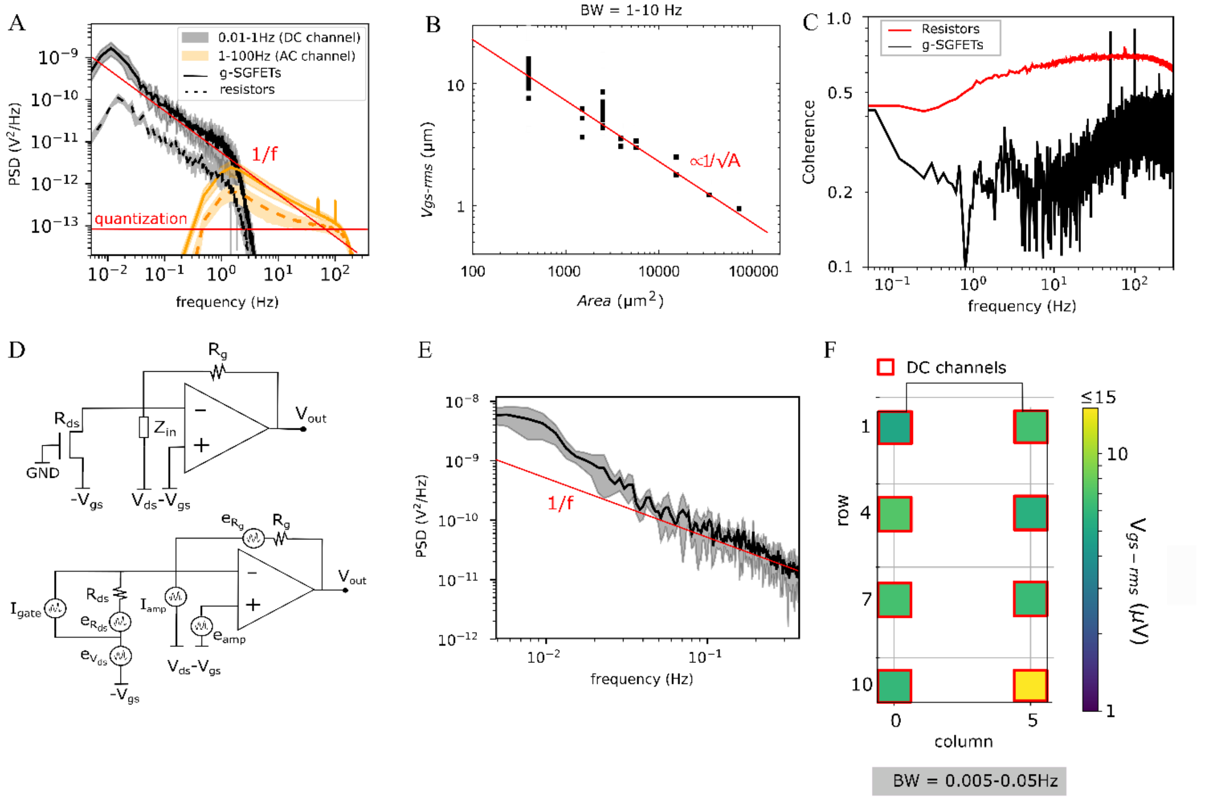

Figure S2. Intrinsic graphene noise and low-frequency contributions from the headstage. (A), Power spectral density of signals from DC-coupled channels (black) and AC channels (orange) measured for g-SGFETs in the beaker (solid) and for 2kΩ resistors (dashed). The red lines indicate the 1/f trend corresponding to intrinsic noise and quantization noise. **(B),** The intrinsic noise of g-SGFETs ($V_{gs\text{-}rms}$ in the 1-10Hz) versus the area of the channel is plotted. The red line indicates the $1/\sqrt{A}$ dependence. **(C),** The coherence for a DC-coupled channel is shown for a resistor (red) and for a g-SGFET (black). **(D),** The equivalent circuit of the g-SGFET connected to the transimpedance amplifier is shown (top) together with the equivalent circuit for the expected noise sources (bottom). **(E),** The power spectral density for the DC-coupled channels with g-SGFETs connected is shown in the infra-slow frequency band. The 1/f trend is indicated by a red line. **(F),** The integrated noise ($V_{gs\text{-}rms}$) in the 0.005-0.05Hz band is shown for all the 8 g-SGFETs connected to DC-coupled channels of the headstage.

Non-coherent noise sources	Coherent noise sources
$V_{out-Rds} = \sqrt{R_{ds}4K_BT}\frac{R_g}{R_{ds}}$	$V_{out-Vds} = e_{vds}\frac{R_g}{R_{ds}}$
$V_{out-Rg} = \sqrt{R_g4K_BT}$	
$V_{out-Iamp} = I_{amp}R_g$	$V_{out-Igate} = g_m e_{gate} R_g$
$V_{out-eamp} = e_{amp}\frac{R_g + R_{ds}}{R_{ds}}$	

Table S2: Output voltage noise produced by coherent and non-coherent noise sources in the amplification chain.

S3. Signal stability

The shift in the CNP presumably caused by adsorption/desorption of charged chemical species on the graphene channel and changes in the reference electrode potential cause very slow drifts of the measured signal. Fig. S3A shows the measured signal *in-vivo* over the first 24h of recording. The observed drift shows an initial rate of ~20mV/h during the first 1-2 hours, which afterwards tends to stabilize. Fig. S3A also shows the high-pass filtered signal above 1mHz, in which the drift is not observed.

Besides long-term changes in the doping of the graphene channel and the reference electrode potential, the reference electrode might present short term instabilities, which could lead to an uncontrolled biasing of the graphene transistor. The use of stainless steel screws to fix the implant on the skull and simultaneously provide a reference potential is a common practice in electrophysiology[3]. However, controlling the bias of active sensors requires the application of a controlled overpotential, in contrast to conventional electrode-based electrophysiological measurements. In this work, it was found that stainless steel screws do not provide a stable reference potential for the operation of g-SGFETs, which might be caused by a poor contact with the dura. In order to circumvent this problem we have used an Ag/AgCl electrode implanted in direct contact with the cerebellum.

The transfer characteristics obtained using a stainless-steel screw in the skull as reference electrode presented inconsistencies for different V_{gs} sweep rates. The stabilization of I_{ds} after changing the gate voltage was very slow causing a defective acquisition of the I_{ds} - V_{gs} curves. Fig. S3C shows the transfer curves for this type of reference electrode obtained by changing V_{gs} at two different rates (2s/step and 40s/step). The differences between the two curves demonstrate that the stationary conditions are not reached. An important drift of the current can be observed which explains the discrepancies between sweep rates in Fig. S3E. In order to eliminate these drifts, an Ag/AgCl electrode was implanted on the cerebellum. Fig. S3B shows the close match between the transfer characteristics obtained for different V_{gs} sweep rates using a Ag/AgCl electrode. Fig. S3D shows the I_{ds} over time during the V_{gs} sweep for this reference electrode, demonstrating a much faster stabilization. These results demonstrate that Ag/AgCl allows to accurately control the bias of the graphene channel during operation *in-vivo*.

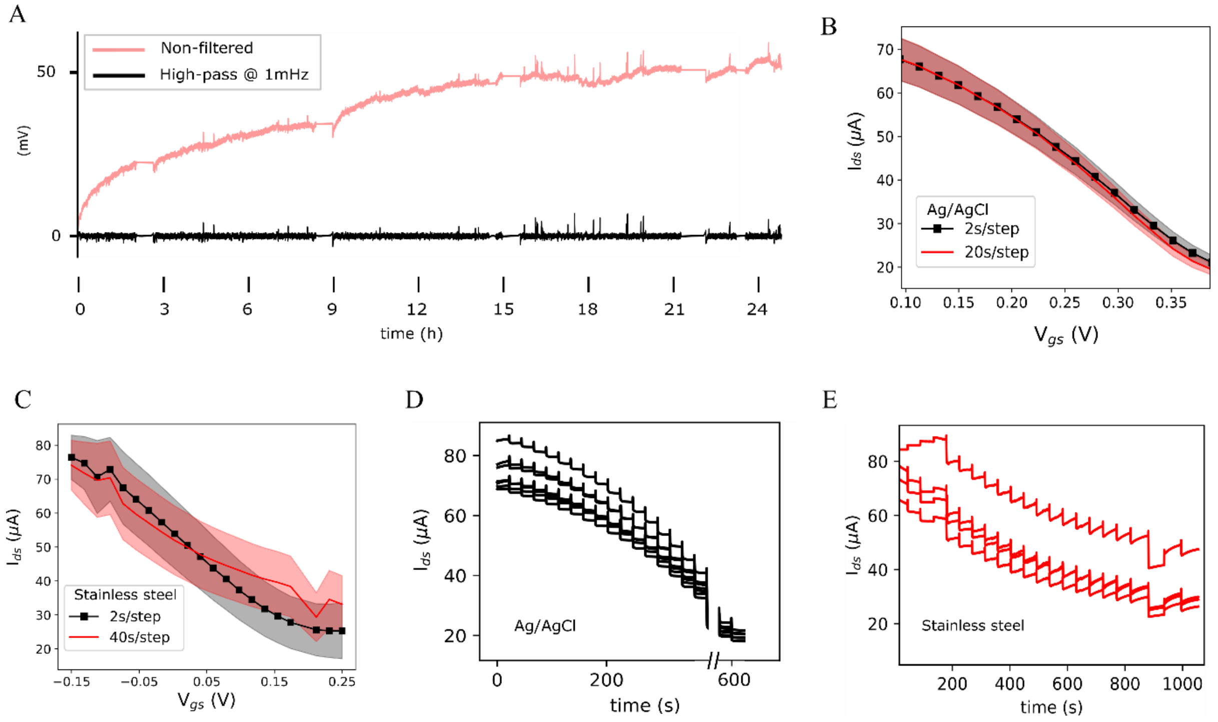

Figure S3. Signal stability. (A), The signal for a DC-coupled channel during the first 24h of recording *in-vivo* is shown in pink and the high pass filtered signal (>1mHz) is plotted in black. **(B),** $I_{ds} - V_{gs}$ curves measured applying two different sweep rates using a Ag/AgCl implanted on the cerebellum (n=7 g-SGFETs). One outlier removed. **(C),** $I_{ds} - V_{gs}$ curves measured applying two different sweep rates using a stainless steel screw implanted on the skull (n=4 g-SGFETs). Solid lines in panels (B) and (C) represent the mean and the error bands the standard deviation. **(D,E),** The I_{ds} over time during a V_{gs} sweep rate of 20s using an Ag/AgCl in contact with the cerebellum **(D)** and a sweep rate of 40s per step and using a stainless steel screw **(E)** is shown. The discontinuity in panel D corresponds to an interruption in the applied voltage (see Source Data file).

S4. Determination of intrinsic noise in g-SGFETs over time

The noise performance of the g-SGFETs is susceptible to degradation over long-term implantation. In order to evaluate the equivalent evolution of intrinsic noise over time the noise power must be measured. Due to the presence of neural activity detected by the g-SGFETs, the *in-vivo* determination of the noise power must be estimated from a frequency band in which, on average, the power of neural activity is below the power of 1/f noise. Fig. S6 shows the PSD of the signal for all the g-SGFETs in the array for day 1 and day 11 after the implantation. It is possible to see that above a certain frequency, the power of neural activity goes below the 1/f from the intrinsic noise of the g-SGFET and the quantization noise from the ADC. In order to determine the evolution of the intrinsic noise of the g-SGFETs we measured the power density at 200Hz over time. Assuming a 1/f response the intrinsic noise of the g-SGFET can be estimated for any frequency band of interest. Note that this noise value will always be equal or larger than the actual intrinsic noise of the g-SGFETs due to possible contributions from the quantization noise or neural signal power. Fig. 3e and 3f of the main text show the V_{gs-rms} and I_{ds-rms} values in the 1-10Hz frequency band estimated following this approach. Note that the V_{gs-rms} values obtained *in-vivo* are close to the ones extracted in PBS, indicating the accuracy of this estimation.

Finally, the orange line in Fig. S4 shows the PSD for a broken g-SGFET. When the transistor degrades and the channel breaks, no current can flow through the transistor. Therefore, the flicker noise from the g-SGFETs does not contribute significantly and only the noise from the amplifiers in open circuit remains (see supplementary information S2). This causes a significant drop in the noise as shown in Fig. S4, which is a clear indicator that the channel has broken. Fig. 3f in the main text shows the yield of g-SGFETs working over time based on this indicator.

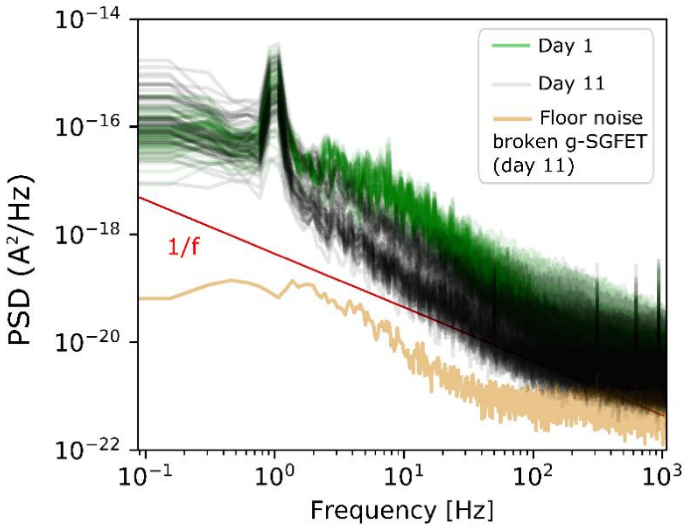

Figure S4. Power spectral density of I_{ds} measured *in-vivo*. The PSD for all 64 g-SGFETs in the array is shown for day 1 and day 11 after the implantation. The red line indicate the 1/f trend. The orange line corresponds to the PSD of a channel which resistance has increased dramatically, probably due to breaking of the graphene sheet.

S5. Frequency response of the g-SGFETs

The magnitude and phase of the transconductance over frequency have been measured *in-vivo.* A certain attenuation of the gain magnitude with an approximately constant slope in a log-log has been observed, which has been previously reported[4]. This slope can be described from the fractional order of constant phase element (CPE) that models the non-ideally capacitive response of the graphene-electrolyte interface. The drop in the magnitude of g_m with frequency presents a $1/f^{1-\alpha}$, where alpha is the ideality factor of the CPE element. This element also introduces a phase delay in the transduced signal of $\pi/2\,(\alpha-1)$, as shown in Fig. S5. Deviations from this model can be observed above $\sim 100Hz$. This positive shift of the phase has been previously reported when capacitive currents from the electrolyte gate to the drain and source of the g-SGFETs occur[4].

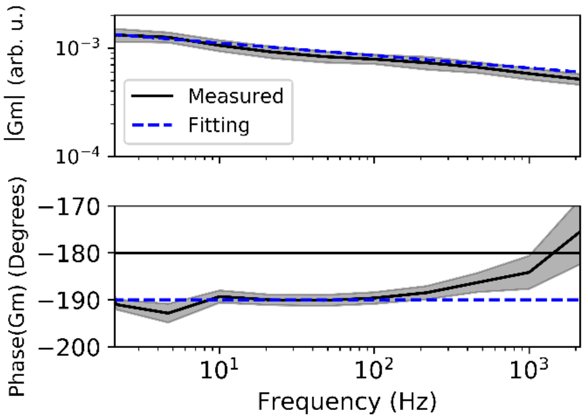

Figure S5. Transconductance over frequency of g-SGFETs in the first day after implantation. The graph on top shows the magnitude of the transconductance while the bottom plot presents the phase of the transconductance (solid line corresponds to the mean and error bands indicate the standard deviation, n=10 g-SGFETs).

S6. Cytokine levels in serum

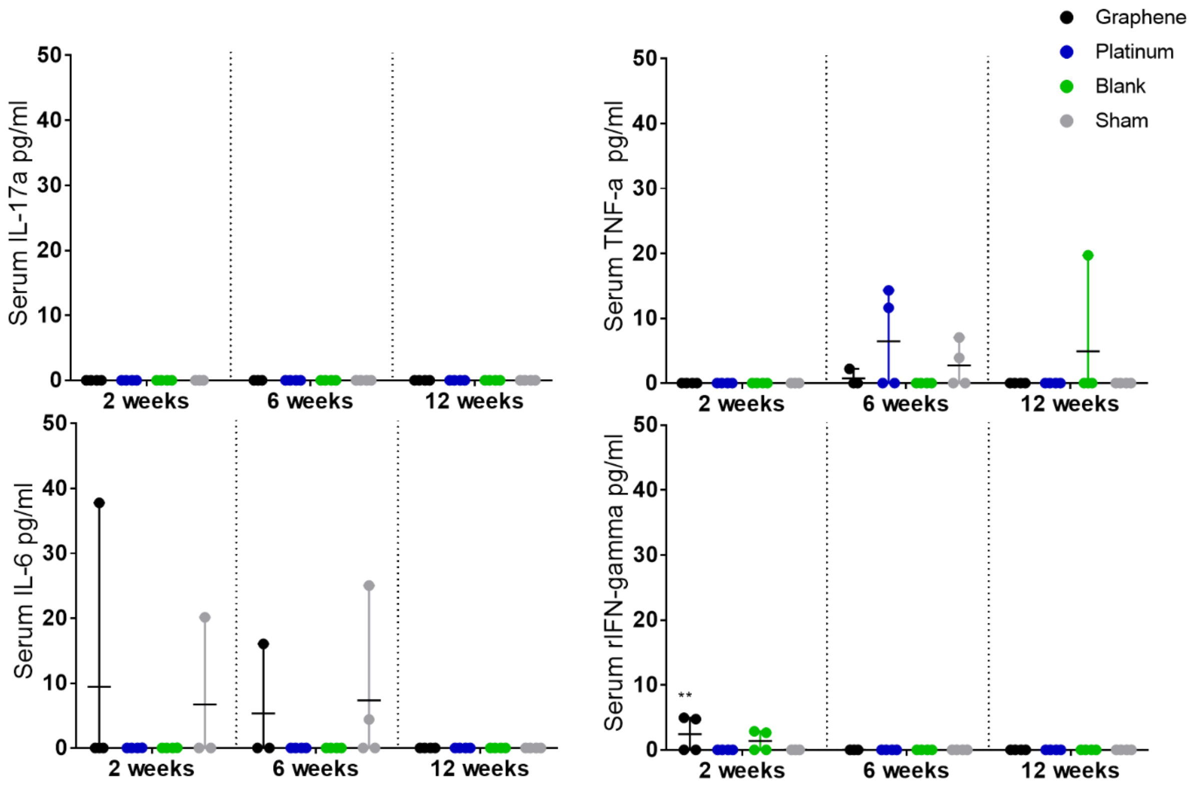

Figure S6. Inflammatory cytokine levels expressed in serum from animals implanted with devices or control animals. Over the four inflammatory markers analysed, IL-17a, TNF-α, IFN-γ and IL-6, there was a significantly higher expression of TNF-α at 2 weeks post implantation for graphene devices compared to sham controls. By 6 weeks this reaction had subsided. No other cytokines showed any significant difference at any timepoint. Bars in panels d and e indicate the mean and range of data point. Two-way ANOVA with Dunnett's multiple comparisons test, compared to sham control for all timepoints. ** indicates p= 0.006.

S7. Cytokine levels in brain tissue

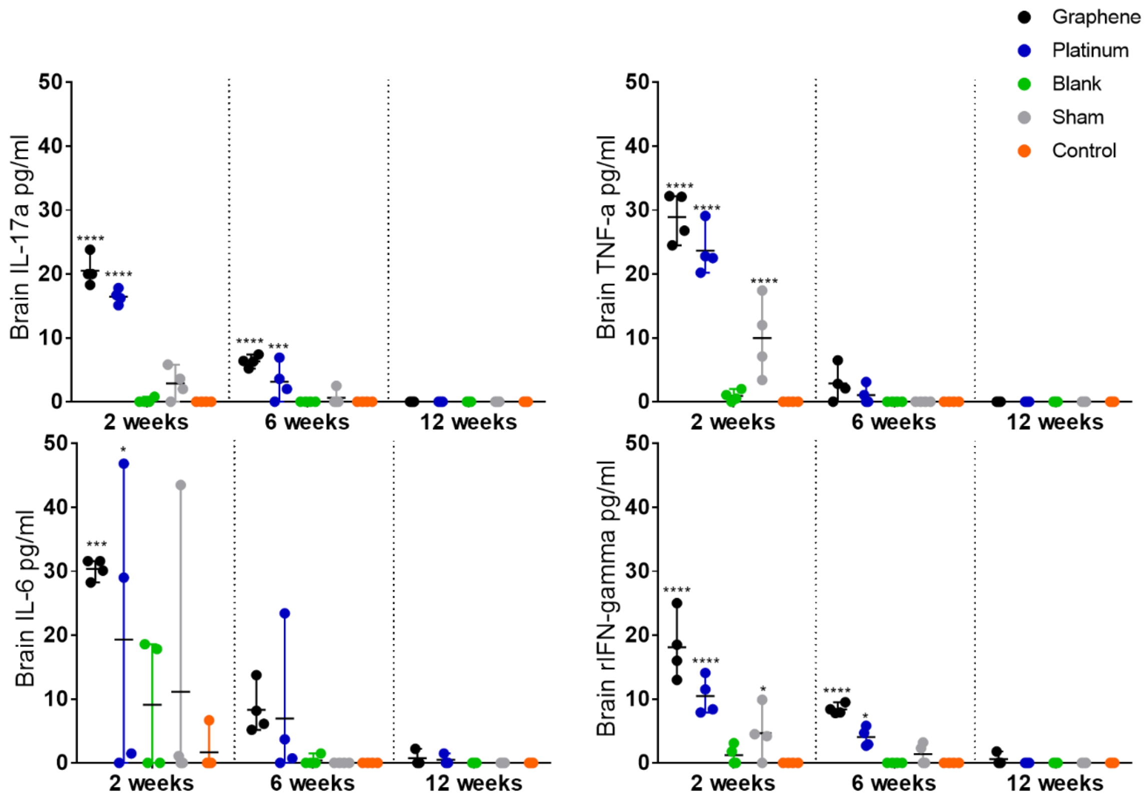

Figure S7. Inflammatory cytokine levels expressed in brain tissue at the surgical site. Over the four inflammatory markers analysed, IL-17a, TNF-α, IFN-γ and IL-6, there was a significantly higher expression at 2 weeks post implantation for graphene and platinum devices compared to naïve animal controls. By 6 weeks, this expression was still significant for both IL-17a and IFN-γ, however by 12 weeks this reaction had subsided. n=4 at 2 and 6 weeks, n=3 at 12 weeks. Bars in panels d and e indicate the mean and range of data point. Two-way ANOVA with Dunnett's multiple comparisons test, compared to naïve control for all timepoints. In all panels, **** indicates p<0.0001. For IL-17a, *** indicates p=0.0016. For IL-6, * and *** indicate p = 0.045 and p = 0.0006 respectively. For rIFN-gamma, * indicates p = 0.012 (for 2 weeks timepoint) and p =0.035 (for 6 weeks timepoint).

S8. Immunoflourescent staining

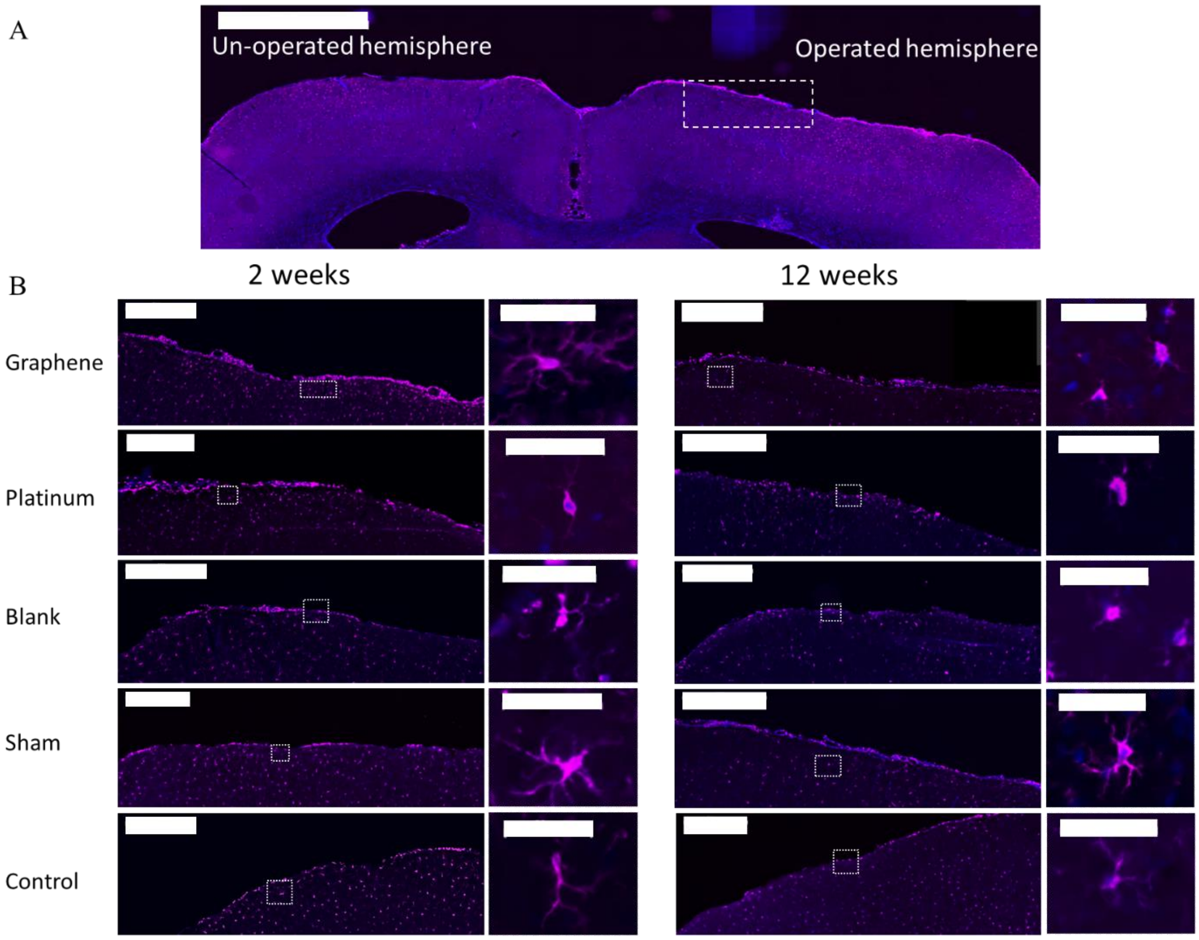

Figure S8. Iba-1 immunoflourescent staining to assess activation status of microglia at the surgical site. (A), Microglial cells were counted for both the operated hemisphere, and the unoperated hemisphere. Scale bar corresponds to 2mm. **(B)**, Exemplar images, taken from the highlighted region in panel A. Left column corresponding to the 2 weeks timepoint and left column to the 12 weeks timepoint. Areas highlighted with white dashed lines are shown with higher magnification. Scale bars correspond to 500µm and 50µm for left and right images respectively within each timepoint.

S9. TUNEL cell counting

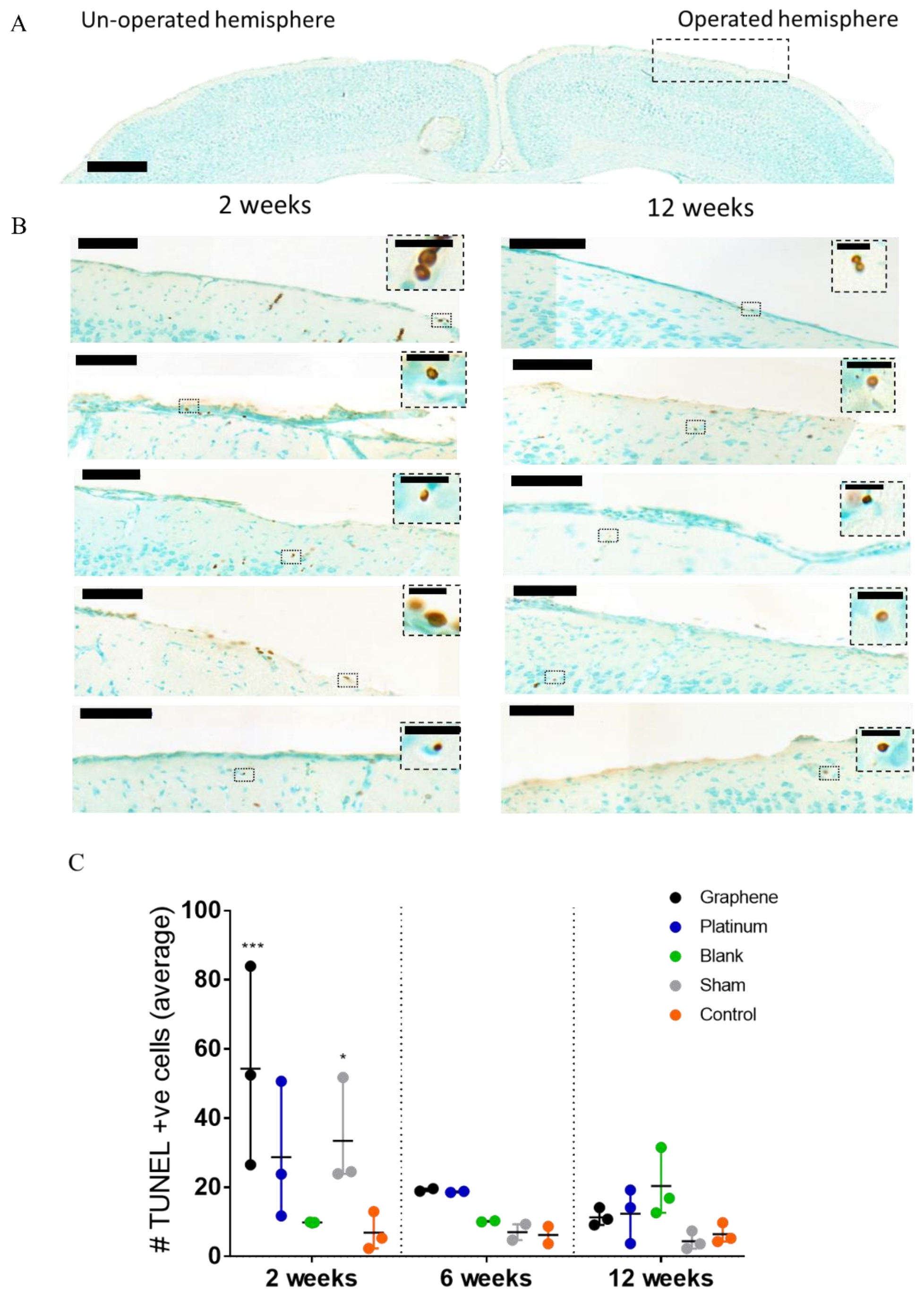

Figure S9. TUNEL positive cell counts from the cortical surface at the implantation site of devices. (A), A gross morphological map of the cortex, outlining where representative images were taken from. Scale bar corresponds to 1mm. **(B)** Representative images of TUNEL positive cells counted across 40 sections of the brain per animal. Scale bar corresponds to 100µm (20 µm for the insets). **(C),** The average counts of TUNEL positive cells at the cortex showed that graphene based devices showed a significant increase in apoptosis in the acute period after implantation, as did the shame operated controls, compared with counts of the contralateral hemisphere. By 12 weeks, there was no significant elevation in apoptosis in the surgical area. n=3 for 2 and 12 week timepoints, n=2 for 6 week timepoints. Bars in panels d and e indicate the mean and range of data point. Two-way ANOVA with Dunnett's multiple comparisons test, compared to contralateral hemisphere for 2 and 12 weeks. * indicates p=0.034, *** indicates p=0.0002,

S10. Rearing event detection

Head elevation of the animal varied widely between 20 and ca 330 mm above ground. The distribution of all head elevation values during the 22h recording displayed marked peaks due to distinct long duration behavioral states (like lying on ground, sitting, walking). In contrast the animal spent only very few time points with its head elevated to more than 200 mm above ground. As can be seen in the time-domain (Fig S10A) these timepoints correspond to repeated very shortlived elevations occurring intermittently during active behavior. Elevations of the head beyond 200 mm from ground were therefore labelled as rearing events.

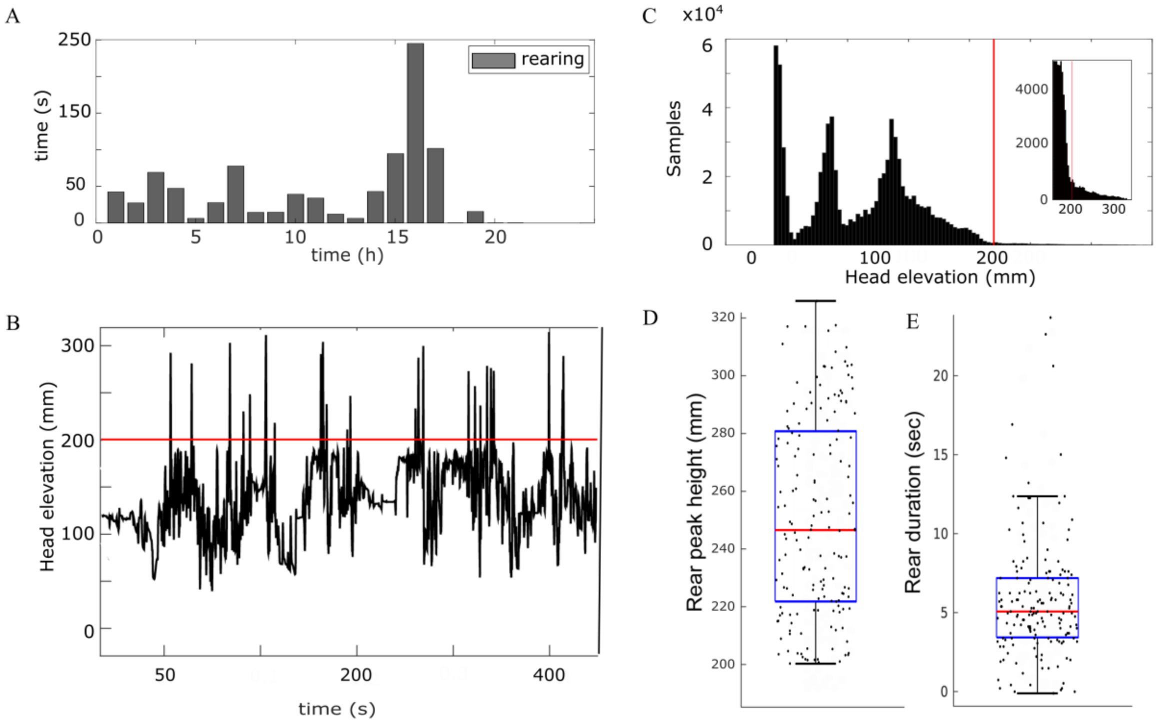

Figure S10. Detection of rearing events. (A), Time spend on rearing over time. **(B),** Example time series of head elevation illustrating the transient and discrete occurrence of rearing events (denoted by *), elevation threshold used for rear detection marked in red. **(C),** Histogram of head elevation during 22h continuous recording period. Threshold for rear event detection marked in red. Zoom-in of same distribution shown in inset. **(D),** distribution of maximum heights for all individual rear events (mean 250,6 ± 2.7 mm). **(E),** distribution of rearing duration for all rear events (mean 5.7 ± 2.8 s). The boxes in panels D and E extend from the lower to the upper quartiles, with a line at the median. The whiskers extend 1.5 times the inter-quartile range and all individual data points are indicated by a dot.

S11. Modulation of high-gamma activity during rearing

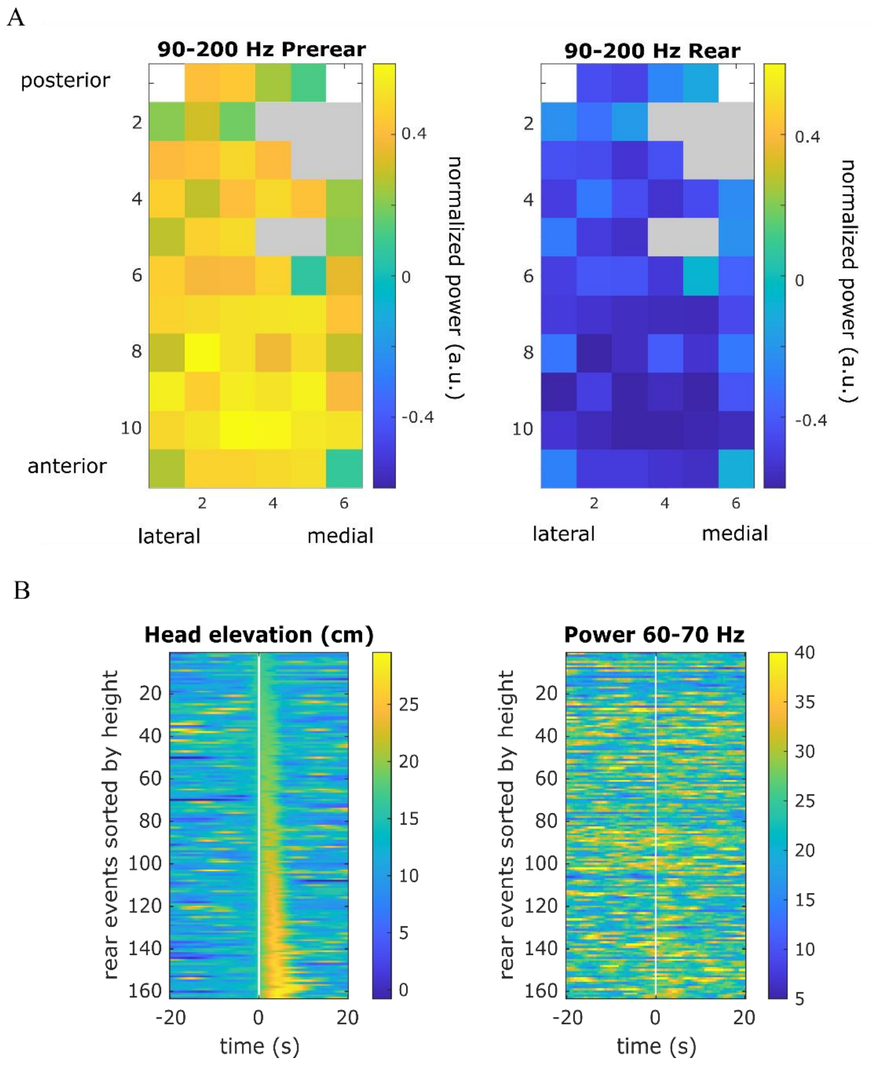

Figure S11. Evaluation of gamma modulation during rearing events. (A), Spatial map of normalized power in the high gamma band for pre-rear (-4s to 0s from rear onset) (left) and rear (0s to 4s from rear onset) (right) **(B)** Head elevation for each rear height event sorted by rear duration (left) and spectral power in 60-70 Hz band for each trial extracted from the same channel as in Fig. 5i, sorted by rear height (right).

S12. Slow and infra-slow power in the SWS-REM transition

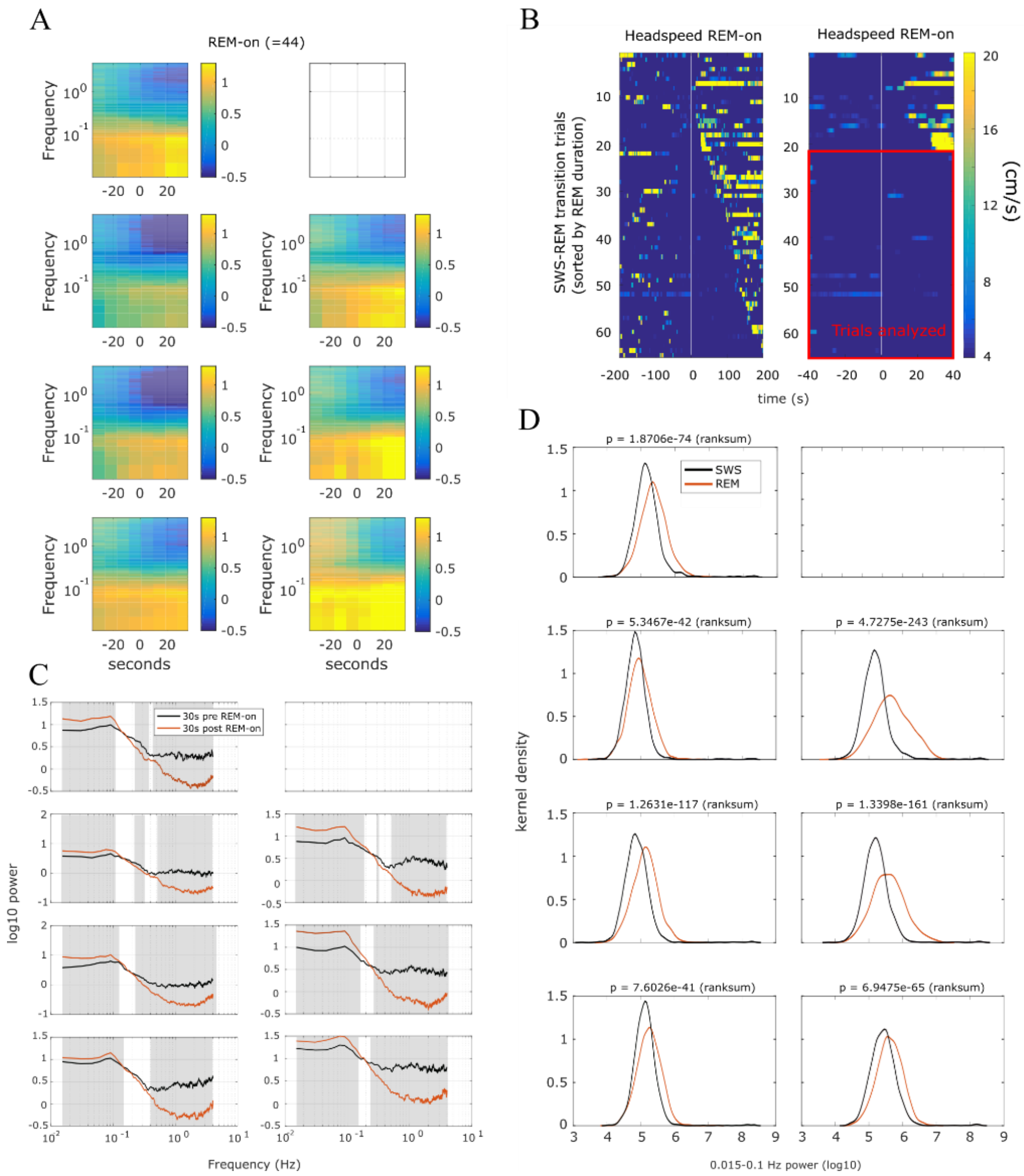

S12. Infra-slow vs slow oscillation band power during SWS/REM sleep: (A), Average spectrogram triggered on REM onsets (n=44) for 7 DC channels with high signal to noise. **(B),** Head speed in the SWS to REM transition for all detected events (left). On the right plot the same data is shown for a shorter time window, where the REM events with duration longer than 40s (n=44) can be identified. **(C),** Median PSD computed for the 30s pre and post REM onsets. Colored area indicates statistically significant difference (two-sided permutation test n=1000 permutations, p<0.05), one channel showing unstable signal is removed. n=44 state transitions. **(D),** Kernel density estimates for the integrated power in 0.01-0.1Hz band during SWS and REM state. p-value for the two-sided Wilcoxon ranksum test are indicated.

References

(1) Schaefer, N.; Garcia-Cortadella, R.; Calia, A. B.; Mavredakis, N.; Illa, X.; Masvidal-Codina, E.; Cruz, J. de la; Corro, E. del; Rodríguez, L.; Prats-Alfonso, E.; et al. Improved Metal-Graphene Contacts for Low-Noise, High-Density Microtransistor Arrays for Neural Sensing. *Carbon N. Y.* **2020**, *161*, 647–655. https://doi.org/10.1016/j.carbon.2020.01.066.

(2) Milotti, E. 1/f Noise: A Pedagogical Review. **2002**.

(3) Gage, G. J.; Stoetzner, C. R.; Richner, T.; Brodnick, S. K.; Williams, J. C.; Kipke, D. R. Surgical Implantation of Chronic Neural Electrodes for Recording Single Unit Activity and Electrocorticographic Signals. *J. Vis. Exp.* **2012**, No. 60. https://doi.org/10.3791/3565.

(4) Garcia-Cortadella, R.; Masvidal-Codina, E.; De la Cruz, J. M.; Schäfer, N.; Schwesig, G.; Jeschke, C.; Martinez-Aguilar, J.; Sanchez-Vives, M. V.; Villa, R.; Illa, X.; et al. Distortion-Free Sensing of Neural Activity Using Graphene Transistors. *Small* **2020**, 1906640. https://doi.org/10.1002/smll.201906640.
